# Hidradenitis suppurativa: key insights into treatment success and failure

**DOI:** 10.1172/JCI186744

**Published:** 2024-11-01

**Authors:** Kelsey R. van Straalen, Vincent Piguet, Johann E. Gudjonsson

**Affiliations:** 1 Laboratory for Experimental Immunodermatology, Department of Dermatology, Erasmus University Medical Center, Rotterdam, Netherlands.; 2 Division of Dermatology, Temerty Faculty of Medicine, University of Toronto, Toronto, Canada.; 3 Division of Dermatology, Department of Medicine, Women’s College Hospital, Toronto, Canada.; 4 Department of Dermatology, University of Michigan Medical School, Ann Arbor, Michigan, USA.; 5 Division of Rheumatology, Department of Internal Medicine, University of Michigan, Ann Arbor, Michigan, USA.; 6 Taubman Medical Research Institute, Ann Arbor, Michigan, USA.

Hidradenitis suppurativa (HS) is a chronic, inflammatory skin disease that affects approximately 1% of the Western population (1). HS is characterized by recurrent painful nodules and abscesses and the formation of epithelialized dermal tunnels in intertriginous areas such as the axillae and groin. In addition to this classic clinical presentation, other less typical lesions, such as comedones, ulcers, and plaques, can occur, and involvement of atypical body sites, including the abdomen, flanks, buttocks, and inframammary regions, is not uncommon (2). Tunnels, also known as sinus tracts, are multiple channels within the dermis or subcutis of various thicknesses, often lined with squamous epithelium, with or without a connection to the skin surface, are frequently regarded as a hallmark of advanced HS. However, it is essential to recognize that not all patients will develop tunnels during their disease course (2). Distinctions in prevalence and clinical presentations have been found across diverse populations, reflecting genetic and environmental differences (2). This heterogeneous clinical presentation has led to multiple clinically and statistically derived phenotype classifications (2). Although these phenotypes are likely driven by underlying differences in pathogenesis and immune profiles, none have been clinically validated or substantiated by genetic or molecular evidence. Consequently, these clinical differences have not been considered in clinical trials or translational HS studies. This incomplete understanding of pathogenesis likely impacts treatment responses in subsets of HS patients and hampers the identification of specific pathways and therapeutic targets. Despite these barriers, the results from recent clinical trials and cellular and molecular studies have crucially shifted our understanding of HS disease pathogenesis, providing insights into treatment successes and failures (Figure 1).

## B cells as a central driver of inflammation

HS lacks a dominant Th cytokine axis, but recent work highlighted the contribution of plasma cells and B cells to the pathogenesis of HS by identifying these cells as the dominant infiltrating leukocytes in late-stage HS lesions, clustering around dermal tunnels (3). Analysis of the signal transduction network within HS lesions showed significant enrichment in B cell receptor, spleen tyrosine kinase (SYK), and Bruton’s tyrosine kinase (BTK) signaling, alongside enriched signals for IL-17A and Toll-like receptors and evidence for increased immunoglobulin production and complement activation. The crucial role of B cells and BTK and SYK signaling in sustaining chronic inflammation is underscored by compelling evidence from a recent 16-week phase II trial of remibrutinib, an oral BTK inhibitor. In this trial, 72.7% of patients treated with remibrutinib achieved the simplified Hidradenitis Suppurativa Clinical Response score (HiSCR) (4). Furthermore, an open-label study involving 20 HS patients treated with fostamatinib, an SYK inhibitor, demonstrated an impressive 85% HiSCR response rate after only 12 weeks (5). While these are small patient numbers and phase II trials, phase III trials are needed to determine whether these approaches have superior outcomes when compared against the 40%–60% response rate among patients on anti-TNF and anti–IL-17 therapies in phase III trials (6, 7).

Importantly, anti-TNF therapy, the first registered biologic treatment for HS, has been shown to primarily target B cell activation, with minimal impact on other inflammatory pathways (8). Immunofluorescence studies revealed that TNF is predominantly localized to the plasma cell and B cell populations in HS skin. Notably, TNF nonresponders exhibited elevated expression of chemokines and receptors involved in myeloid migration, neutrophil chemokines, and B cell–related transcripts (8); these findings were corroborated by another study identifying enriched complement and B cell activation pathways in TNF nonresponders at baseline (9). Finally, rituximab, a monoclonal antibody targeting CD20 present on B cells, has shown some efficacy in small case series (10). Together, these studies underscore the critical role of B cells and their interaction with the TNF pathway in HS pathogenesis, highlighting the potential of a novel nanobody targeting both OX40L and TNF (ClinicalTrials.gov NCT05849922) as a promising therapeutic approach. Of note, OX40L, besides its role in T cell activation, supports T follicular helper (Tfh) cells via its expression on B cells.

While there are currently no data on the exact pathogenic role of B and plasma cells in HS, a wide array of autoantibodies against circulating and tissue antigens have been identified in the lesional skin of HS patients (11). The presence of these autoantibodies, especially in advanced disease stages (Hurley II and III), suggests they form in response to tissue destruction, exacerbated by the externalization of autoantibodies through neutrophil extracellular trap (NET) formation, a hallmark of late-stage disease (12). If pathogenic, they likely contribute to the maintenance and progression of the disease rather than its initiation. This suggests that inhibition of NET formation through a novel monoclonal antibody targeting citrullinated histones H2A and H4 (CIT-013) might yield promising results (13).

Survival of B cells in HS lesional skin is driven by several processes that could differ between disease stages. In nodules and abscesses, neutrophil-derived B cell–activating factor (BAFF) supports B and plasma cell persistence more prominently than in tunnels (14). In this setting, granulocyte colony–stimulating factor (G-CSF) in the presence of bacterial products was the major stimulus for neutrophils’ BAFF secretion. These findings hold promise for a novel anti–BAFF receptor antibody, ianalumab, currently under investigation (ClinicalTrials.gov NCT03827798). While early lesions appear to provide a niche for B cell entry and persistence, it remains uncertain whether these findings truly indicate an early pathogenic disease process or a shift in the inflammatory profile due to the presence of later-stage lesions in other body sites and the recirculation of B cells from these lesions. In contrast with nodules and abscesses, chronic HS lesions show the presence of tertiary lymphoid structures (TLS), driving ongoing cutaneous B cell maturation through class switch recombination and affinity maturation during disease progression (15).

## Prominent role for fibroblasts in inflammation and fibrosis

Both T cells and skin stroma have been found to support TLS formation and facilitate B cell recruitment. Along with peripheral T or Tfh cells, a specific fibroblast subset expressing CXCL13 has been identified in chronic lesional HS skin (15, 16). In-depth analysis of these CXCL13^+^ fibroblasts revealed upregulated pathways involving oncostatin M and IL-17A/F, as well as those attracting and activating neutrophils and lymphocytes (16). TNF was identified as the most prominent cytokine activating CXCL13^+^ fibroblasts. Together with myofibroblasts, these immunologically active CXCL13^+^ fibroblasts were found to demarcate the edges of the inflammatory infiltrate in close spatial proximity to B and plasma cells (16). Indeed, the spatial organization of chronic HS lesional skin has been suggested to loosely reflect an abscess, with neutrophils in and around the lumen of a ruptured tunnel, surrounded by macrophages and T cells, with B and plasma cells situated in the periphery (2, 16). A layer of fibroblasts surrounds this actively inflamed area, potentially to sequester the inflammatory response. Interestingly, SYK inhibitors reduce fibrosis signatures in HS lesional skin (17), and a similar role for BTK inhibitors in attenuating fibrosis has been suggested (18). This highlights the potential for these therapies to modulate immune responses and address fibrosis in HS, offering new hope for effective disease management.

## Bacterial triggers?

The consistent findings of abundant immunoglobulin transcripts, antimicrobial peptides, interferon signatures, B and plasma cells, complement dysregulation, and NETosis could suggest a coordinated effort by the immune system to combat colonizing bacteria within ruptured hair follicles and tunnels. Although bacterial dysbiosis and colonization of tunnels are well-recognized features of HS, the complex interplay between bacteria and the immune system in sustaining and driving the disease and in relation to treatment response remains underappreciated and understudied (19). Specifically, *Prevotella* and *Porphyromonas* species are the most abundant genera present in tunnels (20). *Prevotella* spp. can elicit increased IL-17, TNF, IL-36G, CCL20, and IL-6 expression compared with skin commensals such as *Staphylococcus* *epidermitis* in primary human adult epidermal keratinocytes (21). A recent study revealed that adding clindamycin and rifampicin to adalimumab treatment significantly increased the HiSCR response rate from 41% with adalimumab monotherapy to 86% with combined therapy and significantly reduced the draining tunnel count not captured in the HiSCR (22). These results raise the question of whether the immune response driven by bacterial colonization is a critical factor influencing therapy response.

## Converging on key inflammatory signals and therapeutics

The clinical trial landscape for HS has evolved significantly in recent years, marked by notable successes and unexpected setbacks. One contentious issue is the choice of using as the primary outcome measure HiSCR, which lacks measures for draining tunnels and is plagued by a high placebo response rate, which has been implicated in several trial failures. Nonetheless, a deeper understanding of the results of these single-target treatments within the context of HS pathogenesis offers insights.

Although phase II studies targeting IL-23 achieved HiSCR response rates similar to phase III anti–IL-17 studies (risankizumab 46.8% and guselkumab 50.8% vs. 42% and 45%) (7, 23, 24), they did not meet their primary endpoints. This has raised the question of whether these studies were limited by their primary outcome measures and high placebo rates (38.7% and 41.5% vs. 31%) (7, 23, 24) or if other pathways play a more significant role in the elevated IL-17 levels in HS. While IL-23 is crucial for Th17 cell development, IL-17 is produced by various cells, including γδ T cells, innate lymphoid cells, and neutrophils, through both IL-23–dependent and –independent pathways (25). For example, coculturing naive T cells (along with IL-6 and TGF-β) with in vitro–generated NETs or free histone octamers has been shown to increase IL-17 production in a dose-dependent manner (26), providing an explanation why targeting IL-17 directly, rather than IL-23, might be more effective in HS.

Another promising pathway to target is IL-1 signaling, which has been identified as one of the drivers of the HS lesional transcriptome. However, trials targeting different members of the IL-1 family have demonstrated varying yet mainly disappointing results. Available evidence indicates that IL-1α has only a minor driving role in HS (27), explaining the low response rates in phase II trials of bermekimab (HiSCR; 37.1% and 54.9% vs. 37.1%, and 46.2% on placebo, respectively). Similarly, while IL-36 is significantly upregulated in HS lesional skin, the extent of this upregulation and IL-36G–driven downstream responses seems to be less pronounced than in psoriasis (3), potentially explaining the relatively low HiSCR response rates seen in HS for the anti–IL-36R inhibitors imsidolimab (41%, placebo 35.7%; ref. 28) and spesolimab (38.8%, placebo 34.7%; ref. 29) in phase II trials. Targeting IL-1β has shown more promise both in vitro (27) and in a small 20-patient randomized controlled trial of anakinra, with 78% of patients achieving HiSCR compared with 30% of controls (30). Notably, most of these patients were Hurley stage II, and half were previously treated with anti-TNF therapy. This might hold some clues to the efficacy seen in this clinical trial, as a significantly increased expression of *IL1A* and *IL1B* (trend) has been observed in anti-TNF nonresponders, and IL-1 responses were largely unaffected by anti-TNF therapy (8). In line with this, recent phase II data in HS patients who had previously failed anti-TNF therapy demonstrated a promising clinical response to lutikizumab (HiSCR 48.7%–59.5%, placebo 35%; ref. 31), a monoclonal antibody and dual-variable-domain IL-1α/1β antagonist.

Targeting a broader spectrum of immune responses, including T cells and B cell functions through JAK inhibition, might hold promise for a larger group of HS patients. Recent phase II trials demonstrated a positive trend toward increased clinical response to JAK1 inhibition compared with placebo (brepocitinib, 51.9% vs. 33.3%; povorcitinib, 48.1% vs. 28.8%: and upadacitinib, 38.3% vs. 23.8%) (32, 33, 34).

## Capturing heterogeneity is crucial to unravelling treatment responses

The mixed outcomes of these targeted therapies strongly suggest that we will eventually identify underlying inflammatory endotypes associated with treatment response and specific genotypes and phenotypes. We must start collecting accurate genotypic, phenotypic, and high-resolution baseline lesional transcriptome/proteome data in large-scale clinical HS trials to unravel the heterogeneity driving treatment response. Furthermore, it will enable us to target HS more efficiently in different patient subsets based on sex, ethnicity, and clinical presentations. Combining and comparing these data across trials can pave the way for more effective, targeted, personalized treatment. Novel approaches targeting B cells and dual targets, including B and T cells, might offer the key to the long-sought highly effective treatment modality for HS, alone or in combination with current monoclonals targeting TNF or IL-17.

## Figures and Tables

**Figure 1:**
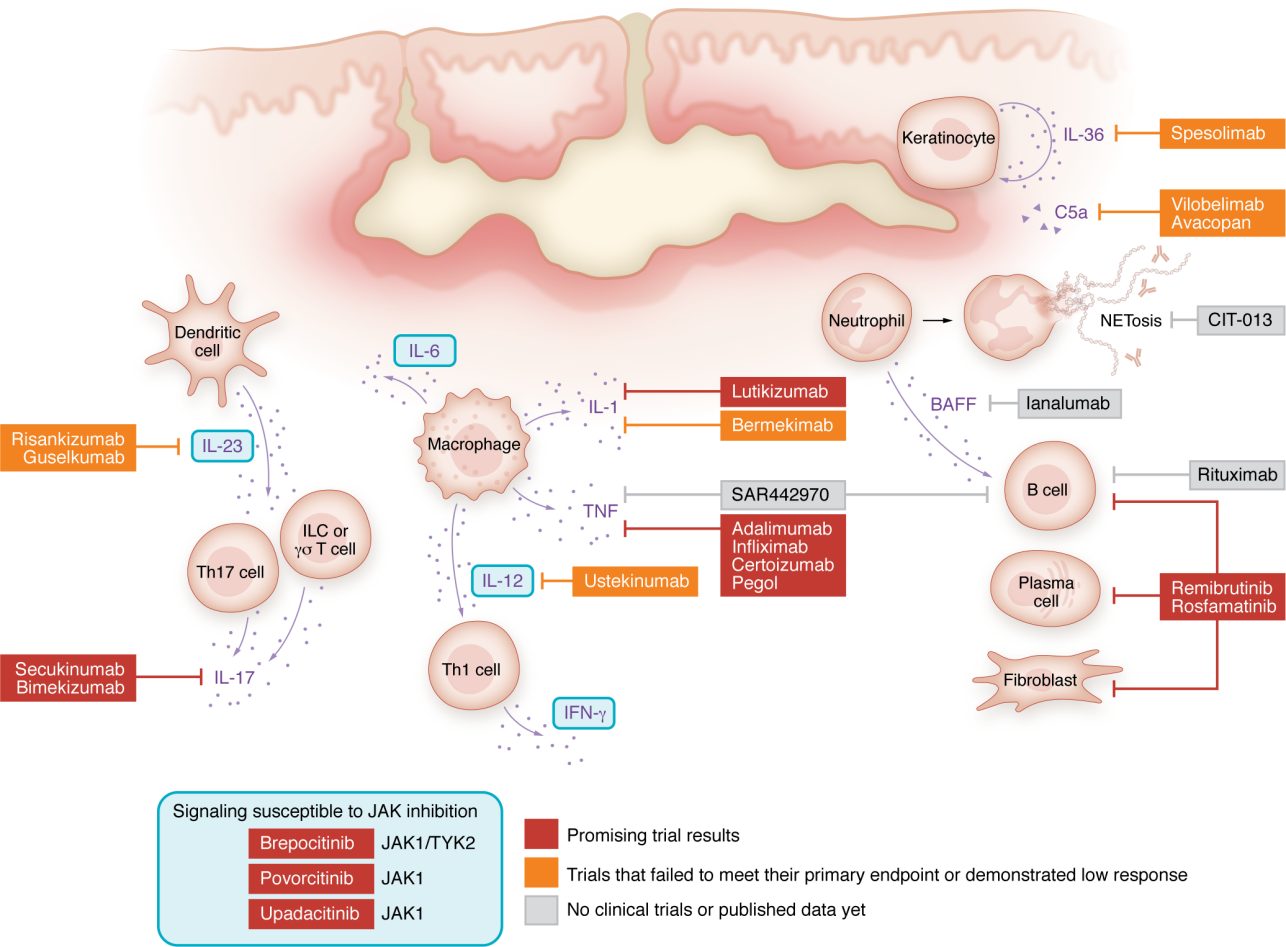
Outline of discussed current and future therapeutics within an active HS lesion. Outline of an active HS lesion with active sinus tracts demonstrating the targets of current and upcoming therapeutics. Colors used for the treatments depicted: red, promising trial results; orange, trails that failed to meet their primary endpoint or demonstrated low response; gray, no clinical trials or published data yet.

## Version 1 11/01/2024

Electronic publication
